# A Rare Presentation of Unicystic Ameloblastoma in an Elderly Edentulous Patient

**DOI:** 10.1155/crid/1622928

**Published:** 2026-06-08

**Authors:** Radha Baral, Samarika Dahal, Dhan Kumari Manandhar, Alina Karna, Ratina Tamrakar, Anumesh Dahal, Krishna KC

**Affiliations:** ^1^ Deparment of Oral Pathology and Forensic Dentistry, Tribhuvan University Teaching Hospital, Kathmandu, Bagmati, Nepal, teachinghospital.org.np; ^2^ Department of Pathology, Tribhuvan University Teaching Hospital, Kathmandu, Bagmati, Nepal, teachinghospital.org.np; ^3^ Department of Oral and Maxillofacial Surgery, Tribhuvan University Teaching Hospital, Kathmandu, Bagmati, Nepal, teachinghospital.org.np

**Keywords:** case report, histopathology, mandible, unicystic ameloblastoma

## Abstract

Unicystic ameloblastoma is a rare, less aggressive variant of ameloblastoma characterized by gross and microscopic cystic appearance. They occur more commonly in young individuals with male predilection. The mandibular molar and ascending ramus region are the common sites of occurrence. Radiographically, unicystic ameloblastoma can present with unilocular or multilocular radiolucency. Three histopathological variants of ameloblastoma have been described in the literature, which are luminal, intraluminal, and mural. This classification is directly related to their biological behavior, surgical management, and prognosis. This case report describes a case of unicystic ameloblastoma of a 78‐year‐old edentulous female patient presented with swelling on the left posterior region of the mandible, which appeared as a cystic lesion radiographically. The lesion was diagnosed as a luminal type of unicystic ameloblastoma after histopathological examination. This report highlights that unicystic ameloblastoma should be taken into consideration while diagnosing cystic lesions occurring in elderly patients.

## 1. Introduction

Ameloblastoma is a slow‐growing, persistent, and locally aggressive odontogenic tumor of epithelial origin [1]. The origin of ameloblastomas is the enamel organ, odontogenic rests of Malassez, reduced enamel epithelium, and lining of the odontogenic cysts [2]. According to the WHO 2022 classification, there are five clinicopathological variants of ameloblastoma, namely conventional, peripheral, adenoid ameloblastoma, metastasizing, and unicystic ameloblastoma (UA) [3].

UA is a rare, less aggressive variant of ameloblastoma characterized by gross and microscopic cystic appearance [4, 5]. They occur more commonly in males, and the mandibular posterior region is the usual site of occurrence [6]. UA is frequently seen in young individuals, more commonly in the second decade of life [4]. Here we report a case of UA in an elderly female in an edentulous mandible.

## 2. Case Report

A 78‐year‐old female patient presented with the chief complaint of pain and swelling in the left lower back region of the jaw for 2 months. The patient was completely edentulous and wearing complete dentures. She also complained of pain and difficulty while wearing dentures. The pain was dull and intermittent in nature. The patient had no medical history. Extraoral examination revealed no gross facial asymmetry, and lymph nodes were also nontender on palpation. Intraoral examination of the patient revealed swelling on the left buccal vestibular region of the mandible extending in the 37 and 38 regions. The overlying mucosa was of normal color. Upon palpation, the lesion was hard and had soft fluctuating areas. The orthopantomogram (OPG) revealed a radiolucent lesion in the second and third molar region extending to the ascending ramus, lined by a well‐defined smooth radiopaque border on the left side of the mandible. The lesion extended from the superior border of the mandible to the near upper margin of the inferior alveolar canal (Figure 1). The aspiration of the cystic content revealed chocolate‐colored viscous fluid. Based upon these findings, differential diagnosis of residual cyst, traumatic bone cyst, UA, and odontogenic keratocyst was given. Enucleation of the lesion was performed, and the cystic sac was removed in toto (Figures 2 and 3). Upon histopathological examination of the lesion, a cystic cavity lined by ameloblastomatous epithelium showing cuboidal to columnar basal cells having hyperchromatic palisading nuclei with reversal of polarization, cytoplasmic vacuolization, and intercellular spacing was revealed. Subepithelial hyalinization could also be appreciated. Superficial cells were stellate reticulum‐like cells (Figure 4). No areas of luminal and mural growth were noted. The connective tissue capsule was composed of dense collagen fibers with a few areas showing inflammatory cell infiltration. The cystic lumen contained cholesterol clefts along with inflammatory exudate. Based on the histopathological findings, the final diagnosis of UA luminal subtype was given. As it was a luminal subtype, which requires only conservative surgical management, no further surgery was done. The patient was kept under follow‐up.

**Figure 1:**
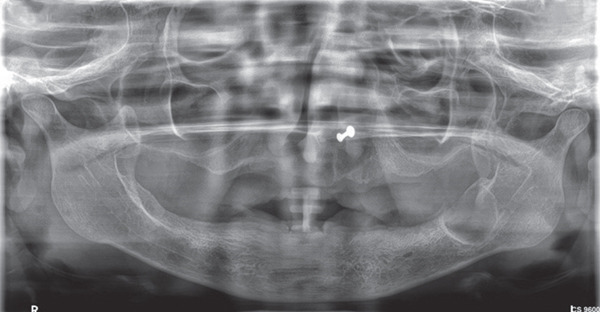
OPG showing unilocular radiolucency with smooth sclerotic border in 37,38 areas.

**Figure 2:**
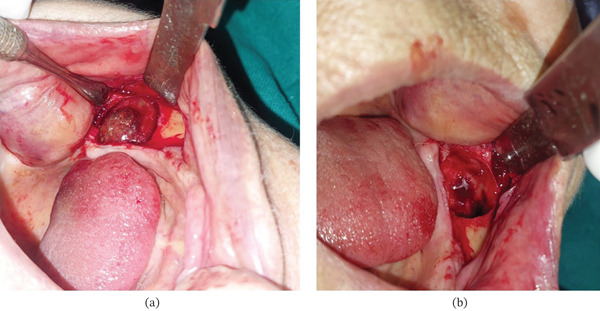
Intraoperative picture. (a) Cystic pathology exposed. (b) The bone cavity remained after enucleation of the lesion.

**Figure 3:**
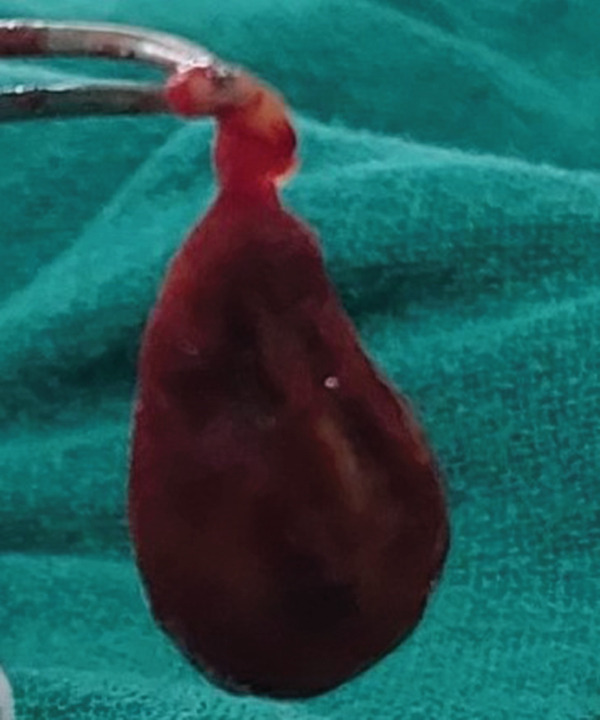
Enucleated cystic sac in toto.

**Figure 4:**
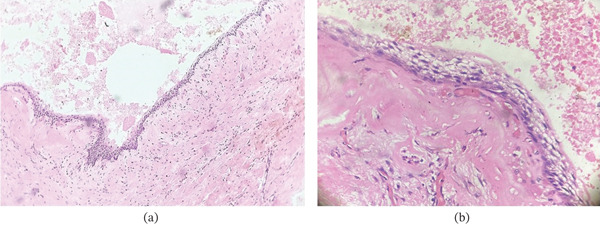
Photomicrographs (a) showing cystic cavity lined by ameloblastomatous epithelium in (hematoxylin and eosin ×40). (b) Cystic content, lining epithelium with subepithelial hyalinization (hematoxylin and eosin ×400).

## 3. Discussion

In 1977, Robinson and Martinez first described UA, which refers to cystic lesions that exhibit clinical and radiologic characteristics of an odontogenic cyst but, on histological examination, show a typical ameloblastomatous epithelium lining part of the cyst cavity, with or without luminal and/or mural tumor proliferation [7]. UA commonly occurs in young individuals as young as the prenatal age [8]. To the best of our knowledge, very few cases have been reported in elderly individuals, and no cases have been reported in edentulous patients [9]. UA is more commonly seen in male patients compared with females [10]. In the present case, the patient was a 78‐year‐old edentulous female. The common site of occurrence of UA is the mandibular molar and ascending ramus, which was also seen in this case [10, 11]. Clinical features of the UA include slow‐growing swelling, locally aggressive in most cases, which was also seen in the present case [4, 6, 9].

Radiographically, UA can present as unilocular or multilocular radiolucency. The term “unicystic” is derived from the gross and microscopic appearance, which is characterized by a cystic cavity lined by ameloblastomatous epithelium; hence, the term “unicystic” should not be confused with the unilocular radiographic appearance, as UA can be multilocular. Radiographic presentation of our case was a well‐defined unilocular radiolucency with a sclerotic border [2, 12].

Three histopathologic variants, namely luminal, intraluminal, and mural types of UA, have been described [2, 5].

The luminal type of UA is restricted to the luminal surface of the cyst. The lesion consists of a fibrous cyst wall, with a lining that comprises partially or totally of ameloblastomatous epithelium. This basal layer consists of columnar or cuboidal cells with hyperchromatic nuclei that show reverse polarity and basilar cytoplasmic vacuolization. The overlying epithelial cells are loosely cohesive and resemble the stellate reticulum [2, 5]. As the histopathology of the present case was consistent with this type of UA, a diagnosis of luminal subtype was given.

Residual cyst, traumatic bone cyst, odontogenic keratocyst, and UA were the differential diagnosis given in the present case, considering the clinical presentation and unilocularity of the lesion in the radiograph.

Traumatic bone cyst was ruled out based on the lack of a scalloped outline on the radiograph. An empty bony cavity without epithelial lining is the diagnostic feature of traumatic bone cyst; in contrast, definite epithelial lining was appreciated in the present case [13].

Residual cyst was a very important differential diagnosis of the present case, as it is interestingly quite common in the edentulous arch and radiographically presents as unilocular, well‐defined radiolucency having a smooth border, as described in the present case. However, histological examination revealed the epithelial lining to be ameloblastomatous epithelium rather than stratified squamous epithelium of a residual cyst [14].

Odontogenic keratocyst was ruled out based on the cystic content, which is usually cheesy white material in odontogenic keratocyst and most importantly, inconsistent histopathological features [15].

Other benign odontogenic tumors were not considered in the differential diagnosis based on the cystic nature of the lesion and the absence of solid tumor areas in histopathology.

Compared with solid multicystic ameloblastomas/conventional ameloblastomas, UAs respond more favorably to conservative management, including enucleation, curettage, and marsupialization [2, 4]. Treatment of the UA varies according to the subtypes; luminal and intraluminal types are treated conservatively, and mural types should be treated in the same manner as conventional ameloblastoma, which requires more aggressive treatment options like marginal or block resection [8, 9]. As the present case was a luminal subtype, enucleation was already done; no further surgical treatment was performed, and the patient was kept under regular follow‐up.

## 4. Conclusions

UA can present as odontogenic and nonodontogenic cysts clinically and radiographically. UA should be taken into consideration while diagnosing cystic lesions occurring in elderly patients.

## Author Contributions

Radha Baral: conceptualization, formal analysis, resources, methodology, writing—original draft, and editing. Samarika Dahal: methodology, supervision, validation, writing—original draft, and editing. Dhan Kumari Manandhar: conceptualization, writing—original draft, writing review and editing, validation, and methodology. Alina Karna: methodology, supervision, validation, and resources, formal analysis. Ratina Tamrakar: methodology, supervision, validation, resources, and data curation. Anumesh Dahal: methodology, supervision, validation, resources, and formal analysis. Krishna KC: methodology, supervision, validation, resources, and validation.

## Funding

No funding was received for this manuscript.

## Disclosure

All authors have read and approved the final version of the manuscript. Corresponding author had full access to all of the data in this study and takes complete responsibility for the integrity of the data and the accuracy of the data analysis.

## Ethics Statement

The authors have nothing to report.

## Consent

Written informed consent was obtained from the patient to publish this report in accordance with the journal′s patient consent policy.

## Conflicts of Interest

The authors declare no conflicts of interest.

## Data Availability

The authors confirm that the data supporting the findings of this study are available within the article.
